# The Role of Immunoglobulin G4 in Outcomes of Primary Sclerosing Cholangitis

**DOI:** 10.3390/jcm13010079

**Published:** 2023-12-22

**Authors:** Miroslav Vujasinovic, Karouk Said, Christina Villard, Jennifer Carlsson, Christopher Poli, Patrick Maisonneuve, J.-Matthias Löhr

**Affiliations:** 1 Department for Upper Abdominal Diseases, Karolinska University Hospital, 141 86 Stockholm, Sweden; karouk.said@akademiska.se (K.S.); matthias.lohr@ki.se (J.-M.L.); 2 Department of Medicine Huddinge, Karolinska Institute, 141 86 Stockholm, Sweden; christina.villard@ki.se (C.V.); christopher.poli@regionstockholm.se (C.P.); 3 Department of Transplantation Surgery, Karolinska Institute, 141 86 Stockholm, Sweden; 4 Faculty of Medicine, Uppsala University, 753 10 Uppsala, Sweden; jennifer.carlsson.6935@student.uu.se; 5 Division of Epidemiology and Biostatistics, IEO, European Institute of Oncology IRCCS, 20141 Milan, Italy; patrick.maisonneuve@ieo.it; 6 Department of Clinical Science, Intervention, and Technology (CLINTEC), Karolinska Institute, 171 77 Stockholm, Sweden

**Keywords:** immunoglobulin, IgG, subclasses, primary, sclerosing, cholangitis

## Abstract

Introduction: Primary sclerosing cholangitis (PSC) is a chronic, cholestatic liver disease that is characterized by an inflammatory and fibrotic process affecting bile ducts which eventually develops into liver cirrhosis and liver failure. The aim of this study was to investigate serum IgG subclass distribution in patients with PSC and its possible association with PSC outcomes. Patients and methods: We performed a retrospective analysis of 181 patients who had been diagnosed with PSC between January 1970 and December 2015 and followed at our outpatient clinic. Their demographic, immunological, and clinical characteristics were recorded and analyzed. Results: This study included 181 patients with PSC (120 males, 61 females). There was no association between IgGs and the development of autoimmune hepatitis, cirrhosis, cholangiocarcinoma, liver transplantation, inflammatory bowel disease, and colectomy. Patients with elevated IgG4 had statistically significant higher rates of cholangitis (*p* = 0.02) and endoscopic retrograde cholangiopancreatography (ERCP) (*p* = 0.009). High IgG4 values were observed in nine patients who underwent ERCP. In these nine patients, on average, IgG4 was evaluated 5 years after ERCP (min 3 days, max 11 years). Subanalysis considering only IgG4 values evaluated before ERCP showed no significant difference but remains significant if we consider IgG4 values after ERCP. Conclusion: Elevated IgG4 in our study showed a possible association with higher rates of cholangitis and ERCP among patients with primary sclerosing cholangitis. It seems that IgGs may be a useful tool for the prediction of outcomes in patients with PSC. A prospective study is necessary, especially to study the trends of IgGs values during disease as well as the role of possible seroconversion.

## 1. Introduction

Primary sclerosing cholangitis (PSC) is a chronic, cholestatic liver disease that is characterized by an inflammatory and fibrotic process affecting bile ducts which eventually develops into liver cirrhosis and liver failure [1]. PSC is classified as a rare condition, affecting fewer than 200,000 individuals in the United States and 250,000 individuals across the European Union; however, there is rising incidence, probably due to changing environmental exposure [2]. Up to 80% of PSC patients have concomitant inflammatory bowel disease (IBD) that can be diagnosed at any time during the course of PSC [1]. Furthermore, PSC is associated with an increased risk of extrahepatic and hepatobiliary malignancies, in particular cholangiocarcinoma [1,3]. Elevated levels of immunoglobulin G (IgG) have been noted in 61% of PSC patients, most often up to 1.5 times the upper limit of normal [4]. A previous study by our study group showed that IgG1 and IgG2 can distinguish patients with immunoglobulin G4-associated cholangitis from those with PSC [5]. Elevated serum IgG subclass (IgGs) levels have been reported in various autoimmune diseases, including primary Sjögren syndrome, systemic sclerosis, and primary biliary cholangitis, showing significantly increased levels of IgG1 and IgG3 compared to those in healthy controls [6]. In other autoimmune diseases, such as systemic lupus erythematosus, differential expression/elevation of IgG subclasses have been reported with significantly higher levels of IgG1, IgG2, and IgG3 compared to healthy controls, but no correlation has been shown between IgG subclasses and distinct clinical features [7]. IgGs are the main isoform of antibodies that can be found in the serum and that serve important protective roles in immunity [8]. The present study aimed to investigate serum IgG subclass distribution in patients with PSC and its possible association with PSC outcomes.

## 2. Patients and Methods

We performed a retrospective analysis of patients who had been diagnosed with PSC and followed at our outpatient clinic at the Department of Upper Abdominal Diseases of Karolinska University Hospital in Stockholm, Sweden. Their demographic, immunologic, and clinical characteristics were recorded and analyzed. 

Laboratory analysis: The analysis of IgGs was performed at Karolinska University Hospital, Laboratory for Clinical Immunology and Transfusion Medicine, using the Optilite system (The Binding Site Group Ltd.; Birmingham, UK) Immunoassay Tests (IgG Kit). The following IgG subclass serum levels were considered normal: total IgG: 6.7–14.5 g/L; IgG1: 2.8–8.0 g/L; IgG2: 1.15–5.7 g/L; IgG3: 0.24–1.25 g/L; IgG4: 0.05–1.25 g/L. 

Inclusion criteria: Patients with PSC age ≥18 years at the time of analysis.

Exclusion criteria: Patients with missing data in medical charts relevant to the present study; patients without a Swedish identity number.

Variables: We analyzed patients’ sex and age at the time of PSC diagnosis and events during follow-up: cholangitis, endoscopic retrograde cholangiopancreatography (ERCP), autoimmune hepatitis (AIH), inflammatory bowel disease (IBD), colectomy, liver cirrhosis, cholangiocarcinoma, and liver transplantation. Patients with events prior to PSC were excluded.

Statistical analysis: IgG levels were categorized as low, normal, or elevated based on laboratory cut-off values and were also described by means and standard deviations. Differences in the distribution of patients’ characteristics across groups were assessed using Fisher’s exact test for categorical variables and Student’s *t* test or ANOVA for continuous variables. The average annual rate of various events was calculated by dividing the number of events observed during follow-up by the number of person-years of observation. The cumulative incidence of liver cirrhosis, cholangiocarcinoma, AIH, IBD, colectomy, and liver transplantation was plotted using the inverse of the Kaplan–Meier method. The log-rank test was used to assess differences in outcomes between groups of patients. Hazards ratios (HRs) and 95% confidence intervals (CIs) were obtained from Cox proportional hazards regression models. The analyses were performed with SAS software version 9.4 (SAS Institute, Cary, NC, USA). All *p*-values were two-sided, and *p*-values less than 0.05 were considered statistically significant.

## 3. Results

**Figure 1:**
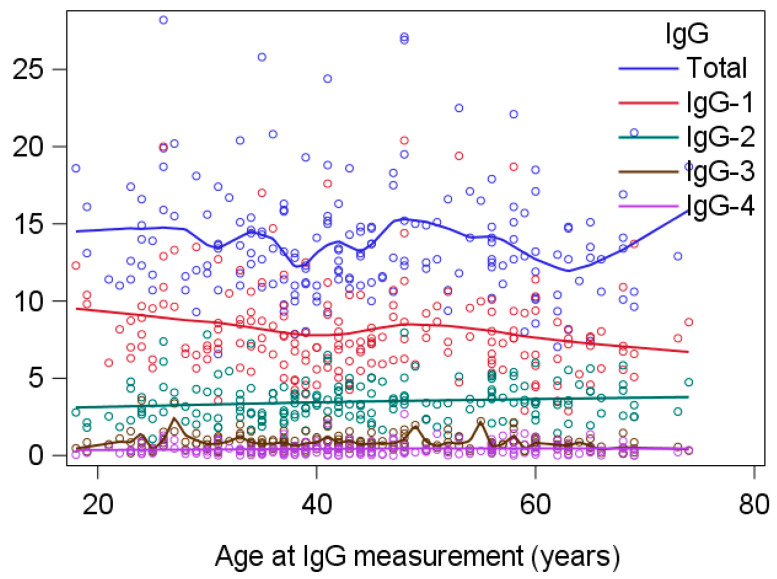
Immunoglobulin subclass (IgGs) levels according to age at diagnosis of primary sclerosing cholangitis (PSC).

This study included 181 patients with PSC (120 males, 61 females) first diagnosed between January 1970 and December 2015. Patients’ demographic, laboratory, and clinical characteristics are presented in Table 1 and Figure 1.

IgG measurement was performed within 5 years of diagnosis of PSC in 58 (32.0%) patients; 5–9 years from diagnosis of PSC in 48 (26.5%) patients; 10–14 years from diagnosis of PSC in 33 (18.2%) patients; 15–19 years from diagnosis of PSC in 20 (11.1%) patients; and ≥20 years from diagnosis of PSC in 22 (12.2%) patients. 

IgG levels were similar in male and female patients with PSC (Table 1). Levels tended to decrease with increasing age at diagnosis of PSC, but without statistical significance. There was no association between IgGs and previous or concomitant diagnosis of IBD (Table 2).

**Figure 2:**
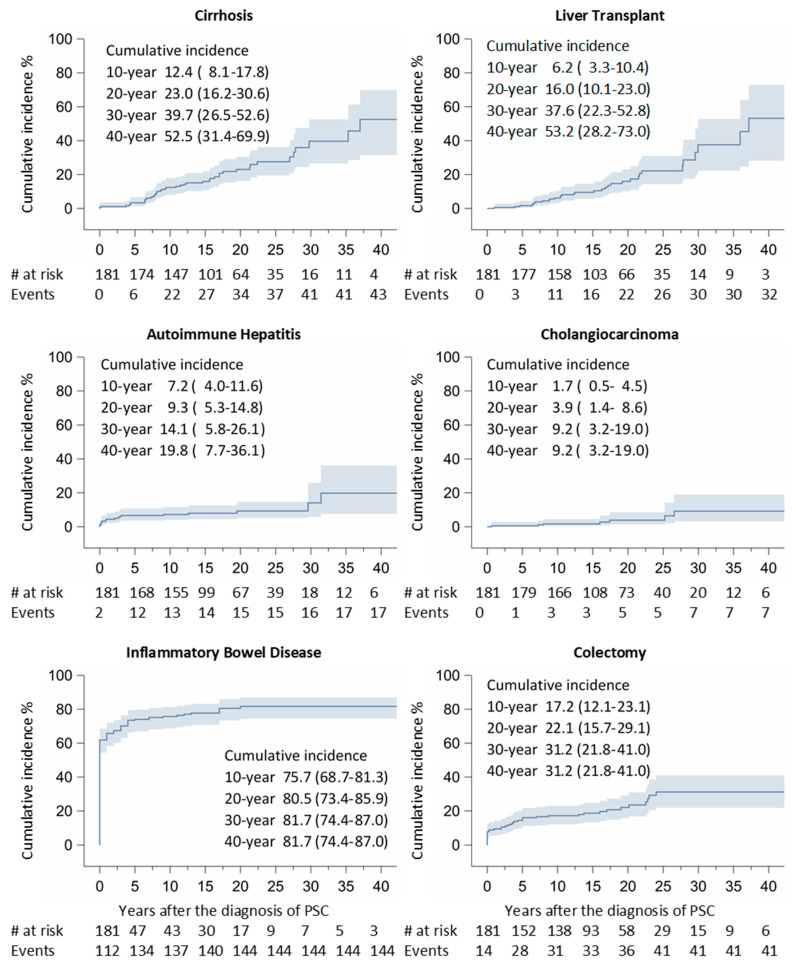
Liver cirrhosis, autoimmune hepatitis, cholangiocarcinoma, IBD, liver transplantation, and colectomy at or after diagnosis of primary sclerosing cholangitis.

During a mean interval of 10.3 years, 45 patients developed liver cirrhosis, 16 developed AIH, 56 developed IBD, 7 developed cholangiocarcinoma, 32 underwent transplantation, and 28 had a colectomy. The cumulative incidence of liver cirrhosis, AIH, IBD, cholangiocarcinoma, liver transplantation, and colectomy is presented in Figure 2.

Table 3 presents the development of various events after the diagnosis of PSC (patients with events prior to PSC were excluded) according to IgG subclasses measured prior to the event. There was no association between IgGs and the development of AIH, cirrhosis, cholangiocarcinoma, liver transplantation, and colectomy.

Patients with elevated IgG4 had statistically significant higher rates of cholangitis (*p* = 0.02) and ERCP (*p* = 0.009). High IgG4 values (>1.25 g/L) were observed in nine (12.6%) patients who underwent ERCP. In these nine patients, in average, IgG4 was evaluated 5 years after ERCP (min 3 days, max 11 years) (Table 4a). Subanalysis considering only IgG4 values evaluated before ERCP showed no significant difference (Table 4b) but remains significant if we consider IgG4 values after ERCP (Table 4c). No association was observed between IgG4/IgG1 or IgG4/IgG ratios and cholangitis and/or ERCP (Table 4).

## 4. Discussion

Previous studies suggested that IgG subclasses may serve as biomarkers for the early diagnosis of liver diseases, including AIH and primary biliary cholangitis (PBC) [8]. In the present retrospective study, serum IgG subclass levels were analyzed to determine their role in the outcomes of patients with PSC. We found no association between IgGs and development of AIH and cirrhosis.

A subgroup of patients with PSC and high IgG4 levels has been recently identified, suggesting a possible distinct clinical phenotype [2]. Many studies have reported elevated serum IgG4 levels (9–27%) in patients with PSC [2,9,10,11,12,13,14,15,16]. However, as emphasized in the recently published European guidelines on IgG4-related digestive diseases, such patients need to be carefully distinguished from patients with IgG4-related cholangitis (biliary manifestation of multi-systemic IgG4 related disease), which is effectively treatable with glucocorticoids and other immunosuppressants [17]. For the same reason, guidelines from the European Association for the Study of the Liver (EASL) on cholestatic liver disease recommend measurement of serum IgG4 levels in all patients with large-duct PSC at diagnosis [1].

A recently published systematic review identified 36 articles that specifically addressed the clinical characteristics of patients with PSC and high IgG4 [2]. Retrospective studies have reported a reduced frequency and more aggressive colitis in PSC with high IgG4 levels compared to normal IgG4 levels [2,9,13]. These results are not confirmed in the present study as we found no association between IgG4 levels and colectomy.

Increased risk of hepatobiliary carcinoma (particularly cholangiocarcinoma and gallbladder carcinoma), as well as increased risk of colorectal cancer in PSC IBD patients, are known in patients with PSC and careful surveillance is necessary [1,2,3]. Previously published studies on PSC and high IgG4 levels have been underpowered to assess the risk of malignancy in this specific cohort of patients [2]. Recent findings indicate that measurement of serum IgG4/IgG1 or IgG4/(IgG1+IgG3) levels may help to differentiate IgG4-related cholangitis from PSC [18]. In the same study, IgG4 alone was shown to be the most accurate serologic marker for the differentiation of IgG4-related cholangitis from cholangiocarcinoma [18]. IgG levels in our study were not associated with the development of cholangiocarcinoma.

A retrospective analysis on 168 patients with PSC showed that prior biliary intervention was more likely in patients with elevated IgG4 levels [11]. However, in the same study (which was published a decade ago), abnormal pancreatic imaging was noted in 15% of patients, meaning (according to current knowledge) that autoimmune pancreatitis type 1 with associated IgG4-related cholangitis should be considered as a possible differential diagnosis in this group of patients [5,17,19,20]. This comment can be generalized to most retrospectively published studies, especially those reporting on the positive effect of glucocorticoid treatment [10,21,22], which is typical for IgG4-related diseases. Patients with elevated IgG4 in our study had higher rates of cholangitis (*p* = 0.02) and ERCP (*p* = 0.009). However, subanalysis considering only IgG4 values evaluated before ERCP showed no significant difference but remains significant if we consider IgG4 values after ERCP. These findings indicate that PSC patients with elevated IgG4 levels may have a more aggressive disease course compared to PSC patients with normal IgG4 levels. The increased incidence of cholangitis in this group of patients indicates that these patients may be at higher risk of developing bile duct strictures requiring therapeutic ERCP. This subgroup of PSC could be a distinct clinical phenotype with a more aggressive course. Unfortunately, there is no current medical therapy that improves survival in patients diagnosed with PSC, and liver transplantation remains the only curative option for progressive disease [2]. The lack of effective medical therapy has resulted in PSC becoming one of the leading indications for liver transplantation in the Nordic countries [23]. Previous studies report contradictory results with regards to disease severity in patients with elevated IgG4 levels and PSC [2,9,10,14,16,24]. Two large retrospective studies showed no association between elevated IgG4 levels and increased risk of liver transplantation and/or liver-related death [14,16], whereas others report more pronounced disease severity in patients with elevated IgG4 levels [2,9,10,24]. The prognostic relevance of serum IgG levels in liver transplantation was investigated in patients with PSC who were followed up for 28 years [25]. The authors identified elevated serum IgG levels at first diagnosis as an independent risk factor for reduced transplant-free survival in patients with PSC [25].

The retrospective nature of this analysis, which is the case in most other previously published articles, is the most important limitation of this study, as well as the fact that IgGs results were measured on a single occasion any time after PSC diagnosis (mean interval 10.3 years after PSC diagnosis). It would be of interest to study the trends and dynamics of IgGs values to see if conversion from seronegative to seropositive values and vice versa (if any) plays a role in PSC outcomes. Recent studies described an association with PSC-high IgG4 and human leukocyte antigen (HLA) class-II haplotypes, T-helper2, and T-regulatory cytokines and chemokines [2]. In our study, potential genetic factors were not evaluated, which is another limitation of this study. However, a large and well-described cohort from a tertiary high-volume center and long follow-up (17 years) are the strengths of this study. Due to the limitations of this study, it is not possible to make strong conclusions on the role of IgG; however, the results of the present study strongly support the current recommendations regarding the control of IgG4 in all patients with PSC. Well-designed prospective multicenter studies with regular control of IgGs are necessary to determine the clinical importance of IgGs in PSC with certainty and usefulness.

## 5. Conclusions

Elevated IgG4 in our study showed a possible association with higher rates of cholangitis and ERCP among patients with primary sclerosing cholangitis. It seems that IgGs may be a useful tool for the prediction of outcomes in patients with PSC. A prospective study is necessary, especially to study the trends of IgGs values during disease as well as the role of possible seroconversion.

## Figures and Tables

**Table 1:** Mean IgGs levels.

	All	Sex		Age at Diagnosis of PSC				
	Patients	Males	Females	0–19	20–29	30–39	40–49	50+
	(*n* = 181)	(*n* = 120)	(*n* = 61)	(*n* = 25)	(*n* = 40)	(*n* = 61)	(*n* = 31)	(*n* = 24)
IgG total (g/L)	13.8 ± 3.7	14.1 ± 3.5	13.2 ± 4.1	14.6 ± 3.3	14.4 ± 3.7	13.5 ± 4.1	13.6 ± 3.3	13.0 ± 3.6
IgG-1 (g/L)	8.1 ± 3.0	8.3 ± 2.8	7.7 ± 3.4	8.9 ± 2.5	8.6 ± 3.1	7.8 ± 3.0	7.8 ± 3.1	7.6 ± 3.1
IgG-2 (g/L)	3.5 ± 1.3	3.6 ± 1.2	3.3 ± 1.4	3.4 ± 1.7	3.4 ± 1.2	3.5 ± 1.3	3.5 ± 1.2	3.6 ± 1.3
IgG-3 (g/L)	0.83 ± 0.52	0.85 ± 0.56	0.80 ± 0.41	1.06 ± 0.82	0.82 ± 0.48	0.83 ± 0.43	0.83 ± 0.48	0.63 ± 0.36
IgG-4 (g/L)	0.43 ± 0.40	0.47 ± 0.44	0.34 ± 0.30	0.34 ± 0.32	0.43 ± 0.38	0.46 ± 0.46	0.42 ± 0.34	0.45 ± 0.43
IgG4/IgG (%)	3.06 ± 2.62	3.24 ± 2.67	2.71 ± 2.53	2.22 ± 1.80	2.88 ± 2.10	3.37 ± 3.06	3.11 ± 2.47	3.42 ± 3.07
IgG4/IgG1 (%)	5.49 ± 5.05	5.75 ± 5.05	4.99 ± 5.06	3.76 ± 1.10	5.07 ± 4.02	6.10 ± 5.89	5.83 ± 5.35	6.02 ± 5.43

Values are expressed as mean ± standard deviation; PSC = primary sclerosing cholangitis; IgGs = IgG subclass. No significant differences according to age or sex.

**Table 2:** Association between IgG and previous or concomitant diagnosis of IBD.

	Total	No Previous IBD	Previous IBD	*p*-Value
ALL	181 (100.0)	93 (100.0)	88 (100.0)	
Sex				
Female	61 (33.7)	33 (35.5)	28 (31.8)	
Male	120 (66.3)	60 (64.5)	60 (68.2)	0.60
Age				
<20	25 (13.8)	18 (19.4)	7 (8.0)	
20–29	40 (22.1)	20 (21.5)	20 (22.7)	
30–39	61 (33.7)	32 (34.4)	29 (33.0)	
40–49	31 (17.1)	15 (16.1)	16 (18.2)	
50+	24 (13.3)	8 (8.6)	16 (18.2)	0.11
IgG (g/L) *				
Mean ± SD	13.8 ± 3.7	13.9 ± 3.6	13.7 ± 3.8	0.69
Low (<6.7 g/L)	1 (0.6)	0 (0.0)	1 (1.1)	
Normal (6.7–14.5 g/L)	118 (65.2)	58 (62.4)	61 (69.3)	
High (>14.5 g/L)	62 (34.3)	35 (37.6)	27 (30.7)	0.39
IgG1 (g/L) *				
Mean ± SD	8.1 ± 3.0	8.3 ± 3.1	7.9 ± 2.9	0.35
Low (<2.8 g/L)	0 (0.0)	0 (0.0)	0 (0.0)	
Normal (2.8–8.0 g/L)	107 (59.1)	53 (57.0)	54 (61.4)	
High (>8.0 g/L)	74 (40.9)	40 (43.0)	34 (38.6)	0.55
IgG2 (g/L) *				
Mean ± SD	3.5 ± 1.3	3.5 ± 1.4	3.5 ± 1.2	0.95
Low (<1.15 g/L)	3 (1.7)	2 (2.2)	1 (1.1)	
Normal (1.15–5.7 g/L)	168 (92.8)	84 (90.3)	84 (95.5)	
High (>5.7 g/L)	10 (5.5)	7 (7.5)	3 (3.4)	0.50
IgG3 (g/L) *				
Mean ± SD	0.83 ± 0.52	0.88 ± 0.53	0.78 ± 0.50	0.19
Low (<0.24 g/L)	10 (5.5)	5 (5.4)	5 (5.7)	
Normal (0.24–1.25 g/L)	145 (80.1)	73 (78.5)	72 (81.8)	
High (>1.25 g/L)	26 (14.4)	15 (16.1)	11 (12.5)	0.81
IgG4 (g/L) *				
Mean ± SD	0.43 ± 0.40	0.46 ± 0.41	0.39 ± 0.39	0.25
Low (<0.05 g/L)	16 (8.8)	5 (5.4)	11 (12.5)	
Normal (0.05–1.25 g/L)	156 (86.2)	82 (88.2)	74 (84.1)	
High (>1.25 g/L)	9 (5.0)	6 (6.5)	3 (3.4)	0.17
				
IgG4/IgG ratio (%) *				
Mean ± SD	3.06 ± 2.62	3.36 ± 2.90	2.75 ± 2.27	0.11
Low (<median 2.40)	91 (50.3)	48 (51.6)	43 (48.9)	
High (>median 2.40)	90 (49.7)	45 (48.4)	45 (51.1)	0.71
IgG4/IgG1 (%) *				
Mean ± SD	5.49 ± 5.05	6.01 ± 5.69	4.95 ± 4.25	0.15
Low (<median 4.06)	91 (50.3)	47 (50.5)	44 (50.0)	
High (>median 4.06)	90 (49.7)	46 (49.5)	44 (50.0)	0.94

IBD = inflammatory bowel disease; IgG = immunoglobulins. * Measured on a single occasion any time after the diagnosis of PSC (mean interval of 10.3 years after diagnosis of PSC (std dev = 8.4 years)).

**Table 3:** Development of various events after the diagnosis of PSC (patients with events prior to PSC were excluded) according to IgG subclasses measured prior to the event.

	Autoimmune Hepatitis	Cirrhosis	Cholangio-Carcinoma	Liver Transplant	Colectomy	IBD
	Events (% per Year)	Events (% per Year)	Events (% per Year)	Events (% per Year)	Events (% per Year)	Events (% per Year)
ALL	4 (6.06)	37 (6.07)	7 (6.93)	29 (6.13)	11 (8.15)	8 (8.16)
IgG (g/L) *						
Low (<6.7 g/L)	-	-	-	-	1 (100.0)	-
Normal (6.7–14.5 g/L)	2 (6.06)	20 (7.55)	6 (8.00)	14 (7.87)	1 (5.26)	6 (8.57)
High (>14.5 g/L)	2 (5.88)	17 (4.91)	1 (3.70)	15 (5.08)	9 (7.83)	2 (7.14)
Log-rank test	0.69	0.06	0.13	0.06	0.007	0.99
IgG1 (g/L) *						
Low (<2.8 g/L)	-	-	-	-	-	-
Normal (2.8–8.0 g/L)	1 (3.33)	18 (7.23)	5 (10.20)	15 (6.49)	3 (11.54)	1 (25.00)
High (>8.0 g/L)	3 (8.11)	19 (5.26)	2 (3.85)	14 (5.79)	8 (7.34)	7 (7.45)
Log-rank test	0.92	0.15	0.04	0.41	0.31	0.008
IgG2 (g/L) *						
Low (<1.15 g/L)	-	1 (11.11)	-	-	1 (100.0)	-
Normal (1.15–5.7 g/L)	4 (6.06)	34 (5.91)	7 (6.93)	28 (6.05)	9 (8.04)	8 (8.16)
High (>5.7 g/L)	-	2 (7.41)	-	1 (9.09)	1 (4.35)	-
Log-rank test	-	0.63	-	0.50	0.006	-
IgG3 (g/L) *						
Low (<0.24 g/L)	-	4 (5.56)	-	2 (9.09)	-	1 (25.00)
Normal (0.24–1.25 g/L)	4 (6.06)	28 (6.18)	5 (10.20)	23 (5.84)	6 (12.24)	7 (7.45)
High (>1.25 g/L)	-	5 (5.88)	2 (3.85)	4 (7.02)	5 (5.81)	-
Log-rank test	-	0.96	0.04	0.43	0.08	0.008
IgG4 (g/L) *						
Low (<0.05 g/L)	-	2 (3.77)	1 (100.0)	3 (6.52)	1 (100.)	-
Normal (0.05–1.25 g/L)	4 (6.06)	31 (6.14)	6 (5.94)	21 (6.05)	10 (7.46)	8 (8.16)
High (>1.25 g/L)	-	4 (7.55)	-	5 (6.25)	-	-
Log-rank test	-	0.52	0.01	0.93	0.002	-
IgG4/IgG (%) *						
Low (<median 2.47)	3 (4.69)	17 (5.25)	6 (6.45)	12 (5.48)	7 (7.07)	2 (7.14)
High (>median 2.47)	1 (50.00)	20 (6.97)	1 (12.50)	17 (6.69)	4 (11.11)	6 (8.57)
Log-rank test	0.08	0.18	0.45	0.56	0.64	0.99
IgG4/IgG1 (%) *						
Low (<median 4.06)	4 (6.06)	19 (5.46)	6 (6.45)	13 (5.70)	8 (7.69)	3 (6.67)
High (>median 4.06)	-	18 (6.87)	1 (12.50)	16 (6.53)	3 (9.68)	5 (9.43)
*p*-value	-	0.28	0.45	0.76	0.86	0.71

* IgG measured at some point before events.

**Table 4:** Association of IgG4 values with cholangitis and ERCP.

a						
	Cholangitis			ERCP		
	No	Yes	*p*-Value *	No	Yes	*p*-Value *
ALL	111	71 (38.8)		78	103 (56.9)	
IgG4 (g/L)						
Low (<0.05 g/L)	8	8 (50.0)		5	11 (68.8)	
Normal (0.05–1.25 g/L)	101	55 (35.3)		73	83 (53.2)	
High (>1.25 g/L)	2	7 (77.8)	0.02	0	9 (100.)	0.009
						
IgG4/IgG (%) *						
Low (<median 2.47)	55	36 (39.6)		40	51 (56.0)	
High (>median 2.47)	56	34 (37.8)	0.81	38	52 (57.8)	0.81
						
IgG4/IgG1 (%) *						
Low (<median 4.06)	42	49 (53.9)		54	37 (40.7)	
High (>median 4.06)	36	54 (60.0)	0.40	57	33 (36.7)	0.58
**b Association of IgG4 values with cholangitis and ERCP (IgG evaluated before ERCP)**						
		ERCP				
		No	Yes	*p*-value *		
ALL		78	32			
IgG4 (g/L)						
Low (<0.05 g/L)		5	2			
Normal (0.05–1.25 g/L)		73	30			
High (>1.25 g/L)		0	0	0.98		
**c Association of IgG4 values with cholangitis and ERCP (IgG evaluated after ERCP)**						
		ERCP				
		No	Yes	*p*-value *		
ALL		78	71			
IgG4 (g/L)						
Low (<0.05 g/L)		5	9			
Normal (0.05–1.25 g/L)		73	53			
High (>1.25 g/L)		0	9	0.002		

* Mantel–Haenszel test for trend.

## Data Availability

The data collected and analyzed during the current study are available from the corresponding author on reasonable request.

## Abbreviations

AIH	autoimmune hepatitis
EASL	European Association for the Study of the Liver
ERCP	endoscopic retrograde cholangiopancreatography
HLA	human leukocyte antigen
IBD	inflammatory bowel disease
IgG	immunoglobulin G
IgGs	IgG subclass
PBC	primary biliary cholangitis
PSC	primary sclerosing cholangitis
